# The Genetic Variability of Members of the SLC38 Family of Amino Acid Transporters (*SLC38A3*, *SLC38A7* and *SLC38A9*) Affects Susceptibility to Type 2 Diabetes and Vascular Complications

**DOI:** 10.3390/nu14214440

**Published:** 2022-10-22

**Authors:** Paolina Crocco, Serena Dato, Alberto Montesanto, Anna Rita Bonfigli, Roberto Testa, Fabiola Olivieri, Giuseppe Passarino, Giuseppina Rose

**Affiliations:** 1 Department of Biology, Ecology and Earth Sciences, University of Calabria, 87036 Rende, Italy; 2 Scientific Direction, IRCCS INRCA, 60121 Ancona, Italy; 3 Clinic of Laboratory and Precision Medicine, IRCCS INRCA, 60121 Ancona, Italy; 4 Department of Clinical and Molecular Sciences, Università Politecnica Delle Marche, 60121 Ancona, Italy

**Keywords:** Type 2 Diabetes, diabetes complications, amino acid transporters, SLC38 family, genetic variability, amino acid availability, amino acid sensing, mTORC1 pathway

## Abstract

Type 2 Diabetes (T2D) is a metabolic disease associated with long-term complications, with a multifactorial pathogenesis related to the interplay between genetic and modifiable risk factors, of which nutrition is the most relevant. In particular, the importance of proteins and constitutive amino acids (AAs) in disease susceptibility is emerging. The ability to sense and respond to changes in AA supplies is mediated by complex networks, of which AA transporters (AATs) are crucial components acting also as sensors of AA availability. This study explored the associations between polymorphisms in selected AATs genes and T2D and vascular complications in 433 patients and 506 healthy controls. Analyses revealed significant association of *SLC38A3*-rs1858828 with disease risk. Stratification of patients based on presence/absence of vascular complications highlighted significant associations of *SLC7A8*-rs3783436 and *SLC38A7*-rs9806843 with diabetic retinopathy. Additionally, the *SLC38A9*-rs4865615 resulted associated with chronic kidney disease. Notably, these genes function as AAs sensors, specifically glutamine, leucine, and arginine, linked to the main nutrient signaling pathway mammalian target of rapamycin complex 1 (mTORC1). Thus, their genetic variability may contribute to T2D by influencing the ability to properly transduce a signal activating mTORC1 in response to AA availability. In this scenario, the contribution of dietary AAs supply to disease risk may be relevant.

## 1. Introduction

Type 2 Diabetes (T2D) is a common metabolic disorder resulting from a combination of inadequate secretion of insulin from β-cells and resistance to insulin action in insulin-dependent tissues such as adipose, liver and muscle tissues [1]. Because of long-term exposure to hyperglycemia, patients with T2D develop microvascular and macrovascular complications, which are the major cause of death in these individuals [2].

Both the incidence and prevalence of T2D are currently growing worldwide [3], and this is likely correlated to the great changes of lifestyles and dietary habits. An inadequate consumption of protein is increasingly recognized as one of the most important nutritional factors related to T2D development [4]. It has been reported, for instance, that a low protein intake during middle age, which leads over time to a reduction in muscle mass, is associated to an increased risk of disease, likely because of the implications on glucose handling by muscle mass, the largest storage depot for glucose in the body [5,6]. On the other hand, it has been documented that a diet relatively high in proteins (especially animal protein), may contribute to disease risk by worsening insulin sensitivity and glucose clearance from the blood [4,7,8,9]. In line with these literature evidence, studies demonstrated that changes in circulating amino acids (AAs) levels, particularly branched-chain AAs (BCAAs, i.e., leucine, isoleucine, valine) are related the development of insulin resistance and T2D risk [10,11,12,13].

Mechanistically, dietary-derived amino acids appear to modulate blood glucose utilization/disposal by acting as mediators of insulin signaling [14]. Recently, amino acids transporters (AATs) have been reported to be crucial components of this regulation as they are responsible for the fine control of cellular and circulating amino acid concentrations. AATs are membrane-bound proteins that act both as carriers and sensors of specific AAs to allow for proper transfer of these nutrients in/out of cells or cellular organelles [15]. They are widely expressed in various tissues and serve diverse functional roles ranging from intracellular energy metabolism to neurotransmission, and anabolic and catabolic reactions [16,17]. Several lines of evidence also indicate that AATs are involved in the regulation of insulin secretion and glucose homeostasis, supporting their contribution to T2D development [18,19,20].

In a previous study we provided some evidence supporting a relationship between the genetic variability of some AATs genes (*SLC7A5*, *SLC7A8*, *SLC36A1*, *SLC38A2*, *SLC3A2*, *SLC38A7* and *SLC38A9*) and the progressive and generalized loss of muscle mass in the elderly, as well as the chance to reach advanced ages, both of which are risk factors for T2D [21]. Thus, based on these findings and the above-mentioned literature evidence, we reasoned that the genetic variability of AAT genes could affects the susceptibility to the T2D and/or its vascular complications. To explore this hypothesis, here we analyzed candidate genetic variants (single nucleotide polymorphisms, SNPs) in AAT genes in a cohort of 939 individuals comprising 433 T2D patients and 506 ethnically matched non-diabetic controls.

## 2. Materials and Methods

### 2.1. Subjects and Measurements

433 unrelated patients diagnosed as affected by T2D (mean age 65.7 ± 7.9 years) and 506 healthy control subjects (the patient’s spouses)) (mean age 58.6 ± 12.3 years) were recruited at the Italian National Research Center on Aging (INRCA) in Ancona (Central Italy).

T2D was diagnosed according to the American Diabetes Association Criteria (American Diabetes Association, 2010). The inclusion criteria and the clinical information collected from each subject were as previously reported in Montesanto et al. [22]. Briefly, demographic, anthropometric, and clinical data were collected for each individual and recorded in a well-defined questionnaire. Measurements of biochemical parameters were undertaken using standardized protocols.

The presence/absence of diabetic complications was established as follows: diabetic retinopathy was defined as dilated pupils evidenced by fundoscopy and/or fluorescence angiography; incipient nephropathy was defined as an urinary albumin excretion rate > 30 mg/24 h and a normal creatinine clearance; chronic kidney disease was defined as an estimated glomerular filtration rate > 60 mL/min per 1.73 m^2^; neuropathy was established by electromyography; ischemic heart disease was defined by clinical history, and/or ischemic electrocardiographic alterations.

Survival data for all cause of mortality were available for patients, gathered during an average of 12 years of follow-up [23].

Ethical approval for this study has been granted by the Ethics Committee of National Institute on Health and Science on Aging (INRCA). All participants gave written informed consent. All methods were performed in accordance with the relevant guidelines and regulations.

### 2.2. SNP Genotyping

For the current study, seventeen SNPs from eight different AAT genes were analyzed, whose basic characteristics are reported in Supplementary Table S1. Candidate genetic variants in AAT genes were selected from a previously study in which significant genetic associations with parameters related to the decline in muscle function and mass in the elderly were identified [21].

DNA was extracted from whole blood (QIAmp 96 DNA Blood kit, QIAGEN). Multiplex SNP genotyping was performed by PCR followed by primer extension and MALDI-TOF mass spectrometry using iPLEX Gold technology from Sequenom (Sequenom Inc., San Diego, CA, USA). Primers for PCR and single base extension were designed by Sequenom MassARRAY Assay Designer software (version 3). The products were PCR amplified using standard procedures, and unincorporated nucleotides were then removed with the shrimp alkaline phosphatase (SAP). Subsequently, a primer extension reaction was implemented using the mass extension primer and the terminator. The primer extension products were then desalted on resin and spotted onto the 384-element SpectroCHIP (Sequenom) for MALDI-TOF analysis using SpectroACQUIRE v3.3.1.3 (Sequenom). Spectra were analysed using MassARRAY Typer v3.4 Software (Sequenom).

For quality control, about ten percent of the analyzed samples was re-genotyped to assess the reliability of the genotype identification protocols. Concordance among duplicates was >99.8% for all SNPs polymorphisms. Additional quality control procedures include the exclusion of SNPs out of the Hardy–Weinberg equilibrium in controls (*p*-value < 0.05) or low genotyping success rates (<90%). Ultimately, three SNPs (rs12794763 in *SLC3A2*, and rs7704138 and rs10056358 in *SLC38A9*) were excluded from analysis.

### 2.3. Statistical Analysis

Continuous variables were presented with mean and standard deviation, and categorical variables were presented with percentage. The Kolmogorov-Smirnov test was used to evaluate the normality of data distribution. Differences in anthropometric and clinical parameters between groups were determined by independent-samples t-test for normal distribution data, the Mann–Whitney U test for skewed data or the χ2 test for categorical variables. For each SNP, departure from Hardy–Weinberg equilibrium was assessed in controls using the χ2 test. Logistic regression models were used to estimate how the variability of analyzed genes influences the predisposition to T2D and related complications. The association analyses were based on the estimation of the odds ratios (ORs) and their 95% confidence intervals (CIs), using age, gender, BMI, and duration of diabetes (when appropriate) as covariates in the formulated regression models.

Additive, dominant, and recessive genetic models were evaluated for each SNP. Akaike’s Information Criterion (AIC) was employed to determine the best-fitting model for each SNP. Only the dominant model was considered where the minor allele homozygote count for either cases or controls was lower than 3%.

To estimate the effect of SNPs on patients’ all-cause mortality, we evaluated survival after 12 years from the baseline visit. Hazard ratios (HR) and 95% confidence intervals (95% CI) were estimated by using Cox proportional hazard models considering age, sex, diabetes duration and complications as confounders.

All statistical analyses were carried out using SPSS software version 27.0 (SPSS, Inc., Chicago, IL, USA). *p* < 0.05 was considered statistically significant. To control for multiple tests, false discovery rate (FDR) correction was applied with a significance threshold (q-value) of 0.05.

## 3. Results

### 3.1. Subject Characteristics

Table 1 outlines the baseline characteristics of T2D patients (with and without diabetes-related complications) and non-diabetic controls. T2D patients were older than control subjects and had a larger proportion of males. As expected, subjects with diabetes differed for all diabetes-related variables, except for insulin levels, and biochemical lipid parameters, when compared with healthy subjects.

### 3.2. Association between AAT Polymorphisms and T2D

As shown in Table 2, significant evidence of association with T2D was detected for rs1858828-G/T in *SLC38A3* gene. Carriers of the TT genotype have approximately a 1.7-fold higher risk of developing T2D than those carrying the G allele (OR  =  1.68, 95% CI: 1.19–2.39; *p* = 0.003). The association remained significant after correction for multiple testing (q-value = 0.046). In support of this result, the same association was replicated when a cohort of self-declared healthy Italians (Tuscans), whose rs1858828 genotypes were retrieved from 1000 Genomes Project (www.1000genomes.org, accessed on 18 July 2022), was used as an additional control group (OR  =  2.27, 95% CI: 2.6–4.10; *p* = 0.0037).

We did not find any evidence of nominally significant genotype-phenotype association when the variability of rs1858828 genotypes was analyzed with respect to the anthropometric and biochemical parameters, both in diabetic and non-diabetic individuals. Since the rs1858828 variant has previously been found associated with the likelihood of reaching very old age, we also assessed the effect of this polymorphism on the risk of all-cause mortality in T2D patients using survival data at 12-year follow-up. However, Cox regression analysis did not show a significant effect of this polymorphism on survival of patients (data not shown).

None of the others analyzed polymorphisms showed association at the nominal threshold of 5%. Yet, a borderline significant decreased risk of T2D was observed for SNPs rs731710-A/G (*SLC7A5*) and rs3783436-T/C (*SLC7A8*) under a dominant model (OR  =  0.73, 95% CI: 0.51–1.03; *p* = 0.076 and OR  =  0.70, 95% CI: 0.48–1.04, *p* = 0.078, respectively).

### 3.3. Association between AAT Polymorphisms and T2D Complications

Nearly 50% of T2D patients showed at least one of the five diabetes-related micro-macrovascular complications analyzed in this study. Among these patients, 120 (27.7%) had one, 66 (15.2%) had two, 19 (4.4%) had three, 13 (3%) had four and 1 (0.2%) had five. In Table S2 are reported the demographics and clinical characteristics of T2D patients with and without vascular complications. Table S3 lists the results of the association test for each SNP, while Table 3 summarizes the associations significant at the 0.05 threshold. Specifically, homozygous carriers of the minor rs3783436-T/C allele in *SLC7A8* and carriers of the minor allele of rs9806843-A/G in *SLC38A7* gene were at lower risk of retinopathy (OR  =  0.17, 95% CI: 0.03–0.93; *p* = 0.016, and OR 0.51, 95% CI: 0.27–0.97; *p* = 0.039 respectively). Moreover, we found that the presence of the minor allele of rs4865615-G/C in *SLC38A9* conferred a protective effect against risk of chronic kidney disease (OR = 0.170, 95% CI: 03–0.82; *p* = 0.012).

## 4. Discussion

Herein, we explored the association between variants of some candidate AATs genes and risk of T2D and related complications. We identified a risk SNP (rs1858828) in *SLC38A3*, a gene belonging to the sodium-coupled neutral amino acid transporters (SNAT) of the SLC38 (System *n*) family and highly expressed in liver, kidney, and brain, but also in pancreas and skeletal muscle. Specifically, an increased risk of diabetes was found among homozygous carriers of the minor T allele of rs1858828, which lies at 291bp 5′ of *SLC38A3* gene in a DNase I hypersensitive region that alters a binding site for Yin Yang 1 (YY1), a dual function transcription factor involved in T2D [24,25], as suggested by inspection of databases providing functional annotation of SNPs.

The SLC38A3 encoded protein, also known as SNAT3, mainly mediates both the import and export, depending on cellular demand, of glutamine, a conditionally essential amino acid under metabolic and nutritional stress conditions [26,27]. Glutamine is the most abundant free amino acid in human body, involved in many fundamental biological functions such as protein and glutathione synthesis, energy production, maintenance of optimal antioxidant status, and immune function [28].

Several evidence support a role for SLC38A3 in glucose homeostasis. SLC38A3 induction mediates glutamine transport in β-cells of pancreas [29], and its downregulation was found in islets of diabetes-prone db/db mice [30]. Studies reported that elevated plasma levels of glutamine induce the uptake of this AA by SLC38A3 with consequent stimulation of insulin secretion through the conversion of glutamine to glutamate [27,31]. Conversely, when plasma levels of glutamine are low it is released by SLC38A3 and taken up by α-cells inducing the release of glucagon and the increase of hepatic gluconeogenesis [32,33], a process substantially increased in patients with T2D [34]. Hepatic gluconeogenesis is inhibited by insulin, a process that does not work properly when insulin signaling is compromised, such as insulin resistance in T2D [35]. Interestingly, the expression of SLC38A3 was found downregulated by chronic treatment of insulin in hepatocytes via the insulin-activated PI3K-mTOR signaling pathway [36]. To this regard, it is worth remarking that glutamine is one of the AA that activate the nutrient-sensitive kinase mammalian target of rapamycin complex 1 (mTORC1), the major signaling hub that integrates different inputs, such as nutrients, energy, and growth factors, to regulate diverse cellular functions [37]. mTORC1 signaling activity was found significantly increased in islets/β cells from patients with T2D [38], and, more recently, it was shown to modulate β-cell mass and function [39]. In this respect, the marginal association of the allelic variants *SLC7A5* rs731710-G and *SLC7A8* rs3783436-C with a reduced risk of diabetes is a result that deserves some attention. These two genes encode for plasma membrane sodium-independent AA antiporters, named LAT1 and LAT2 respectively, typically mediating the simultaneous efflux of glutamine out of cells and uptake of extracellular leucine, the primary essential AA that activates protein synthesis in skeletal muscle through activation of mTORC1 [40,41]. Leucine also acts at multiple points along the insulin signaling and glucose metabolism pathways [42,43] playing a role on diabetes development [44,45]. Interestingly, perturbed levels of both glutamine and leucine are reported in people with T2D with respect to non-diabetic subjects [11,12]. It is also interesting to note that in elderly subjects the same allelic variants of *SLC7A5*-rs731710 and *SLC7A8*-rs3783436 were found associated with better physical performance [21], reported to be impaired in people with diabetes [46]. Overall, results so far indicate that genetic variability of AAT genes may contribute to diabetes development, most likely by affecting mTORC1 activity in multiple metabolic organs such as the pancreas, liver, and skeletal muscle.

mTORC1 signaling pathway has been also related to the pathophysiology of diabetes complications [47]. Interestingly, we found the rs3783436 variant at *SLC7A8* gene associated to a reduced risk of diabetic retinopathy, the most common diabetic eye disease leading cause of vision loss among adult people [48]. Of note, dysfunctional SLC7A8 has been associated with formation of cataract [49], a relatively frequent side effect in people with diabetes [50]. A protective effect against retinopathy was also observed for the intronic SNP rs9806843 in *SLC38A7* gene which encodes a sodium-coupled AAT, known also as SNAT7, which has glutamine as the preferred substrate. Hägglund et al. [51] reported that *SLC38A7* has a relatively broad expression, with high levels in both glutamatergic and GABAergic neurons, and that it may have an important function in the reuptake and recycling of glutamate in these cells. This proposed role of SLC38A7 provides a possible mechanism on which to base the association between the rs9806843 variant of this gene and retinopathy we found. Indeed, glutamate acts as an excitatory neurotransmitter in the retina, yet an excess of glutamate may be toxic to retinal neurons by overactivation of glutamate receptors [52].

Finally, the *SLC38A9* rs4865615-C variant was significantly associated with a decreased risk of chronic kidney disease. SLC38A9 (SNAT9) is a sodium coupled AAT located in lysosomes which acts as an arginine sensor to activate mTORC1 [53] and mediates the efflux of most essential AAs from lysosomes, including leucine, in an arginine-regulated manner [54]. mTORC1 plays an important role in the regulation of renal cell homeostasis and in the development of several kidney disorders [55]. On the other hand, kidney plays a central role in arginine metabolism, being the proximal tubule a major site of arginine production from citrulline [56]. Because rs4865615 is a missense mutation (p.Ser182Thr), it could impact on SLC38A9 function with potential consequences for mTORC1 activity.

## 5. Conclusions

In summary, data herein provide the first evidence, to our knowledge, that sequence variations in AAT genes may affect the risk of developing T2D and related outcomes. Importantly, the susceptibility genes we identified encode for carriers, but overall, for sensors of AAs, specifically glutamine, leucine, and arginine, directly connected with the activation of the major nutrient signaling pathway mTORC1. The positive link between elevated mTORC1 activity in presence of AA overload and diabetes is now recognized and supported by several studies (for a recent review see [57]. The mTORC1-centric paradigm of T2D proposed by Bar-Tana [58] points indeed to mTORC1 as an upstream driver that may generate the multiple disease aspects of T2D. It is, therefore, reasonable to hypothesize that the variability of the above AAT genes can induce changes in the activation of mTORC1, by affecting the capability to transduce a signal in response to AA availability. Consequently, alteration and disturbance of diabetes-related metabolic traits, such as glucose metabolism, insulin resistance, and β-cells functioning may occur.

It is clear, though, that validation of the current results in an independent set of patients is necessary to conclude on a relationship between variability at the AAT loci and T2D susceptibility, and this is a limitation of our study. Also, the elucidation of the functional aspects of the identified SNPs can shed some light on the molecular signaling events or mechanisms behind the genetic associations found. Finally, since some research has suggested that a high intake of dietary proteins may have negative effects on T2D occurrence, it would be of interest to investigate possible effects of SNP–diet interactions on T2D risk. A correlation, if any, between the presence of risk variants and inadequate amino acid intake from dietary proteins could be useful for personalized nutritional therapy that considers personal genotype. Insight into all these aspects could suggest new avenues of investigation.

## Figures and Tables

**Table 1:** Clinical and biochemical characteristics healthy control subjects and patients with type 2 diabetes (T2D).

Characteristics	Controls (*n* = 506)	T2D (*n* = 433)	*p*
*Sociodemographic factors*			
Age (mean, SD)	58.6 (12.3)	65.7 (7.9)	<0.001
Gender (males, % )	207 (40.9%)	244 (56.4%)	<0.001
*Diabetes-related variables*			
BMI, kg/m^2^ (mean, SD)	27.2 (4.8)	28.8 (4.4)	<0.001
WHR (mean, SD)	0.88 (0.08)	0.93 (0.07)	<0.001
HOMA-IR (mean, SD)	1.56 (1.47)	2.91 (2.8)	<0.001
Fasting Glucose, mg/dL (mean, SD)	93.2 (10.1)	162.9 (47.5)	<0.001
HbA1c, % (mean, SD)	5.65 (0.4)	7.44 (1.3)	<0.001
Insulin, µIU/mL (mean, SD)	6.71 (5.4)	7.06 (5.4)	0.32
Age at onset (years)	-	51.8 (11.5)	
Durations of diabetes (years)	-	13.7 (12.3)	
*Biochemical variables*			
Total cholesterol, mg/dL (mean, SD)	215.5 (37.4)	207.1 (37.8)	0.001
LDL cholesterol, mg/dL (mean, SD)	123.9 (31.9)	117.1 (32.2)	0.001
HDL cholesterol, mg/dL (mean, SD)	57.5 (14.3)	51.9 (14.2)	<0.001
Triglycerides, mg/dL (mean, SD)	103.4 (69.5)	139.2 (94.0)	<0.001
*Complications*			
Retinopathy (*n*, %)	-	110 (25.4)	
Neuropathy (*n*, %)	-	75 (17.3)	
Nephropathy (*n*, %)	-	52 (12)	
Chronic Kidney Disease (*n*, %)	-	14 (3.2)	
Ischemic heart disease and stroke (*n*, %)	-	76 (17.6)	

Abbreviations: SD, standard deviation; BMI, Body Mass Index; WHR, waist-to-hip ratio; HOMA-IR, homeostasis model assessment of insulin resistance; HbA1c, glycosylated haemoglobin; LDL-C, low-density lipoprotein cholesterol; HDL-C, high-density lipoprotein cholesterol.

**Table 2:** Association analysis of AATs SNPs with Type 2 Diabetes.

Gene	SNP	Major/Minor Allele	MAF	OR (95% CI) ^a^	Pets Model
*SLC3A2*	rs12804553	G/T	0.30	0.86 (0.62–1.20)	0.38 ^D^
	rs4726	C/T	0.21	0.91 (0.64–1.29)	0.59 ^D^*
*SLC7A5*	rs4329925	T/C	0.15	1.10 (0.77–1.58)	0.59 ^D^*
	rs731710	A/G	0.42	0.73 (0.51–1.03)	0.076 ^D^
*SLC7A8*	rs999165	T/A	0.24	0.89 (0.61–1.30)	0.55 ^D^*
	rs12588118	C/G	0.23	1.35 (0.67–2.72)	0.39 ^R^
	rs3783436	T/C	0.27	0.70 (0.48–1.04)	0.078 ^D^
*SLC36A1*	rs357618	A/G	0.32	0.78 (0.45–1.37)	0.39 ^R^
	rs357629	A/G	0.34	0.82 (0.46–1.45)	0.49 ^R^
	rs14160	T/C	0.14	0.72 (0.47–1.10)	0.12 ^D^*
*SLC38A2*	rs1873793	T/C	0.45	1.14 (0.77–1.69)	0.50 ^D^
*SLC38A3*	rs1858828	G/T	0.43	1.68 (1.19–2.39)	0.0033 ^R^
*SLC38A7*	rs9806843	A/G	0.40	1.20 (0.85–1.69)	0.30 ^D^
*SLC38A9*	rs4865615	G/C	0.26	1.56 (0.84–2.90)	0.16 ^R^

Abbreviation: MAF, Minor Allele Frequency; OR, odds ratio; CI, confidence interval. OR adjusted for age, sex, BMI. ^a^ The best genetic-effect model for each SNP on diseases risk was estimated based on the smallest Akaike’s information criterion (AIC) value. ^D^ is dominant model (T2D risk in heterozygotes plus minor allele homozygotes relative to common allele homozygotes) and ^R^ is recessive model (T2D risk in minor allele homozygotes relative to common allele homozygotes plus heterozygotes). * For these SNPs, only the dominant model was considered since the rare homozygous genotype was <3%.

**Table 3:** SNPs associated with diabetic microvascular and macrovascular complications.

Diabetic Complication	Gene	SNP (Allele)	OR (95% CI) ^a^	*p*-Value
Retinopathy	*SLC7A8*	rs3783436 (T/C)	0.17 (0.03–0.93) ^R^	0.016
	*SLC38A7*	rs9806843 (A/G)	0.51 (0.27–0.97) ^D^	0.039
Chronic Kidney Disease	*SLC38A9*	rs4865615 (G/C)	0.17 (0.03–0.82) ^D^	0.012

Abbreviation: OR, odds ratio; CI, confidence interval. OR adjusted Age, sex, BMI, and diabetes duration were included as covariates. ^a^ The best genetic-effect model for each SNP was estimated based on the smallest Akaike’s information criterion (AIC) value. ^D^ is dominant model (risk of diabetic complication in heterozygotes plus minor allele homozygotes relative to common allele homozygotes) and ^R^ is recessive model (risk of diabetic complication in minor allele homozygotes relative to common allele homozygotes plus heterozygotes).

## Data Availability

The data that support the findings of this study are available from the corresponding author upon reasonable request.

## Supplementary Materials

The following supporting information can be downloaded at: https://www.mdpi.com/article/10.3390/nu14214440/s1, Table S1. Basic characteristics of the candidate genes and selected SNPs. Table S2. Demographics and clinical characteristics of T2D patients with and without micro- and macrovascular complications. Table S3. Association analysis of candidate SNPs with diabetic microvascular and macrovascular complications.
