# Supplementary material for: The Genetic Variability of Members of the SLC38 Family of Amino Acid Transporters (SLC38A3, SLC38A7 and SLC38A9) Affects Susceptibility to Type 2 Diabetes and Vascular Complications
Source: Nutrients. 2022 Oct 22;14(21):4440. doi: 10.3390/nu14214440 (PMC9654215; doi:10.3390/nu14214440)
Supplement: Supplementary file 1 [file nutrients-14-04440-s001.zip › nutrients-1970515-supplementary.pdf]

Table S1. Basic characteristics of the candidate genes and selected SNPs.

Genes	Aliases	Chr	SNPs	Major/Minor allele	Mutation Type
SLC3A2	CD98	11	rs12804553	G/T	Intron variant
			rs4726	C/T	Synonymous variant
			rs12794763	T/G	Intron variant
SLC7A5	LAT1	16	rs4329925	T/C	3'near gene variant
			rs731710	A/G	Intron variant
SLC7A8	LAT2	14	rs999165	T/A	Intron variant
			rs12588118	C/G	Intron variant
			rs3783436	T/C	Intron variant
SLC36A1	PAT1	5	rs357618	A/G	Intron variant
			rs357629	A/G	Intron variant
			rs14160	T/C	3′UTR variant
SLC38A2	SNAT2	12	rs1873793	T/C	Intron variant
SLC38A3	SNAT3	3	rs1858828	G/T	5′near gene variant
SLC38A7	SNAT7	16	rs9806843	A/G	Intron variant
SLC38A9	SNAT9	5	rs4865615	G/C	Missense variant
			rs7704138	C/T	Intron variant
			rs10056358	A/T	Intron variant

Table S2. Demographics and clinical characteristics of T2D patients with and without micro- and macrovascular complications.

	**Retinopathy**			**Neuropathy**			**Nephropathy**			**Chronic Kidney Disease**			**Ischemic heart disease and stroke**		
	Presence	Absence	*p*	Presence	Absence	*p*	Presence	Absence	*p*	Presence	Absence	*p*	Presence	Absence	*p*
Age (Mean, SD)	67.1 (7.8)	65.2 (7.8)	0.032	66.1 (6.7)	65.6 (8.1)	0.61	66.5 (9.2)	65.6 (7.7)	0.49	71.9 (5.4)	65.5 (7.9)	0.001	67.4 (6.4)	65.4 (8.1)	0.021
Sex (Males, %)	58.2	55.9	0.73	73.3	52.9	0.001	71.2	54.5	0.025	78.6	55.7	0.11	68.4	53.9	0.022
Insulin, μIU/mL (Mean, SD)	6.2 (5.03)	7.3 (5.49)	0.05	6.5 (4.1)	7,2 (5,6)	0.21	8.3 (6.6)	6.9 (5.2)	0.15	11.4 (9.9)	6.9 (5.1)	0.16	7.3 (5.7)	7.0 (5.3)	0.64
Age at onset (years)	46.05 (11.8)	53.97 (10.58)	<0.001	47.4 (11.5)	52.8 (11.2)	<0.001	51,7 (13.5)	51.8 (11.2)	0.97	51.3 (13.4)	51.8 811.4)	0.89	51.7 (12.7)	51.8 (11.2)	0.93
Durations of diabetes (years)	21.3 (12.08)	11.2 (9.4)	<0.001	18.7 (11.9)	12.54 (12.1)	<0.001	14.7 (12.1)	13.6 (12.3)	0.54	21.0 (13.3)	13.4 (12.2)	0.066	15.7 (12.8)	13.2 (12.1)	0.13
BMI, kg/m^2 (Mean, SD)	27.8 (3.1)	29.1 (4.7)	0.001	28.9 (3.9)	28.7 (4.5)	0.69	29.2 (4.9)	28.7 (4.3)	0.49	28.9 (3.0)	28.7 (4.4)	0.98	29.0 (4.1)	28.7 (4.5)	0.55
WHR (Mean, SD)	0.93 (0.06)	0.92 (0.06)	0.71	0.95 (0.06)	0.93 (0.07)	0.024	0.95 (0.06)	0.93 (0.07)	0.14	0.96 (0.05)	0.93 (0.07)	0.08	0.94 (0.07)	0.93 (0.07)	0.31
Fasting glucose (mg/dL) (Mean, SD)	176.4 (51.1	158.3 (45.4)	0.001	179.6 (53.5)	159.4 (45.5)	0.003	184.2 (52.4)	160.0 (46.2)	0.002	176.4 (47.8)	162.5 (47.5)	0.31	170.7 (52.0)	161.3 (46.5)	0.14
HbA1c % (Mean, SD)	7.8 (1.1)	7.3 (1.2)	<0.001	7.8 (1.2)	7.3 (1.2)	0.003	7.9 (1.5)	7.3 (1.2)	0.022	7.7 (1.6)	7.4 (1.2)	0.53	7.6 (1.5)	7.4 (1.2)	0.20
HOMA-IR (Mean, SD)	2.7 (2.3)	2.9 (2.9)	0.40	2.9 (2.2)	2.9 (2.9)	0.94	3.8 (3.2)	2.8 (2.7)	0.042	5.1 (4.6)	2.8 (2.7)	0.10	3.3 (3.0)	2.8 (2.7)	0.25
TC, mg/dl (mean, SD)	202.3 (39.3)	208.6 (37.2)	0.14	200.3 (36.8)	208.5 (37.9)	0.087	208.6 (38.3)	206.8 (37.8)	0.76	211.6 (28.3)	206.9 (38.1)	0.55	194.0 (37.3)	209.8 (37.4)	0.001
LDL-C mg/dl (mean, SD)	113.1 (35.5)	118.3 (30.8)	0.17	111.3 (33.3)	118.2 (31.8)	0.10	117.9 (38.9)	116.9 (31.2)	0.86	107.1 (32.1)	117.4 (32.1)	0.29	101.3 (29.5)	120.4 (31.7)	0.001
HDL-C, mg/dl (mean, SD)	52.1 (14.6)	51.8 (14.2)	0.87	49.3 (15.9)	52.4 (13.8)	0.12	46.2 (12.9)	52.6 (14.3)	0.001	44.4 (15.2)	52.1 (14.2)	0.08	48.9 (15.2)	52.5 (14.0)	0.059
Triglycerides, mg/dl (mean, SD)	142.1 (106.2)	142.5 (106.2)	0.97	145.3 (108.2)	141.8 (116.8)	0.80	176.1 (106.5)	137.8 (115.7)	0.019	228.3 (143.2)	139.5 (113.3)	0.038	173.5 (108.1)	135.8 (87.1)	0.11

Abbreviations: SD, standard deviation; BMI, Body Mass Index; WHR, waist-to-hip ratio; HbA1c, glycosylated haemoglobin, HOMA-IR, homeostasis model assessment of insulin resistance; TC, total cholesterol; LDL-C, low-density lipoprotein cholesterol; HDL-C, high-density lipoprotein cholesterol.

Table S3. Association analysis of candidate SNPs with diabetic microvascular and macrovascular complications.

		Retinopathy		Neuropathy		Nephropathy		Chronic Kidney Disease		Ischemic heart disease and stroke	
**Gene**	**SNP (Alleles)**	**OR (95% CI)[a]**	***p***	**OR (95% CI)[a]**	***p***	**OR (95% CI)[a]**	***p***	**OR (95% CI)[a]**	***p***	**OR (95% CI)[a]**	***p***
SLC3A2	rs12804553 (G/T)	1.41 (0.80-2.50) [D]	0.23	0.57 (0.31-1.06) [D]	0.074	1.49 (0.73-3.02) [D]	0.27	1.53 (0.38-6.24) [D]	0.55	1.98 (0.75-5.20)[R]	0.18
	rs4726 (C/T)	0.99 (0.54-1.81) [D*]	0.96	1.50 (0.79-2.87) [D]	0.22	0.72 (0.33-1.61) [D]	0.42	0.47 (0.09-2.39) [D*]	0.34	1.23 (0.66-2.28) [D*]	0.51
SLC7A5	rs4329925 (T/C)	0.86 (0.46-1.61) [D]	0.63	1.08 (0.56-2.08) [D]	0.81	1.27 (0.60-2.70) [D]	0.54	2.35 (0.58-9.57) [D*]	0.24	1.18 (0.62-2.23) [D*]	0.61
	rs731710 (A/G)	1.32 (0.68-2.54) [D]	0.41	0.78 (0.40-1.51) [D]	0.47	1.12 (0.50-2.54) [D]	0.70	0.41 (0.10-1.68) [D]	0.23	0.71 (0.38-1.34) [D]	0.3
SLC7A8	rs999165 (T/A)	1.30 (0.62-2.71) [D]	0.49	0.85 (0.38-1.90) [D]	0.69	2.28 (0.89-5.88) [D*]	0.085	1.07 (0.16-7.33) [D*]	0.95	1.16 (0.50-2.66) [D*]	0.73
	rs12588118 (C/G)	0.66 (0.37-1.20) [D]	0.17	0.92 (0.48-1.77) [D]	0.80	1.12 (0.54-2.34) [D]	0.76	0.57 (0.13-2.57) [D*]	0.46	1.20 (0.65-2.23) [D]	0.56
	**rs3783436 (T/C)**	**0.17 (0.03-0.93) [R]**	**0.016**	1.23 (0.59-2.56) [D]	0.59	1.17 (0.49-2.77) [D]	0.72	1.04 (0.19-5.57) [D]	0.90	1.55 (0.74-3.24) [D]	0.25
SLC36A1	rs357618 (A/G)	1.72 (0.72-4.11) [R]	0.23	1.48 (0.58-3.81) [R]	0.43	0.50 (0.24-1.04)[D]	0.061	0.38 (0.09-1.57) [D]	0.17	0.64 (0.35-1.17) [D]	0.15
	rs357629 (A/G)	1.82 (0.75-4.39) [R]	0.19	1.33 (0.72-2.47) [D]	0.36	0.56 (0.27-1.15) [D]	0.11	0.37 (0.1-1.51) [D]	0.15	0.68 (0.37-1.23) [D]	0.20
	rs14160 (T/C)	1.04 (0.42-2.56) [D]	0.94	0.43 (0.13-1.39) [D]	0.13	0.73 (0.23-2.34) [D]	0.59	0.5 (0.11-1.48) [D]	0.41	0.63 (0.24-1.67) [D]	0.34
SLC38A2	rs1873793 (T/C)	0.65 (0.36-1.19) [D]	0.17	1.49 (0.75-2.98) [D]	0.25	1.83 (0.81-4.15)[R]	0.16	0.48 (0.05-4.28) [D]	0.48	1.36 (0.69-2.67) [D]	0.36
SLC38A3	rs1858828 (G/T)	0.90 (0.5-1.63) [R]	0.73	0.68 (0.38-1.22) [D]	0.2	1.41 (0.69-2.88) [R]	0.36	1.09 (0.28-4.16)[R]	0.27	0.65 (0.37-1.15) [D]	0.14
SLC38A7	**rs9806843 (A/G)**	**0.51 (0.27-0.97) [D]**	**0.039**	1.59 (0.77-3.31) [D]	0.2	1.70 (0.66-4.35)[R]	0.29	0.41 (0.07-2.46)	0.32	1.60 (0.71-3.59)[R]	0.27
SLC38A9	**rs4865615 (G/C)**	0.77 (0.43-1.39) [D]	0.39	0.71 (0.37-1.36) [D]	0.31	0.69 (0.33-1.45) [D]	0.33	**0.17 (0.03-0.82)** [D]	**0.012**	0.76 (0.40-1.43) [D]	0.4

Abbreviation: OR, odds ratio; CI, confidence interval. OR adjusted Age, sex, BMI and diabetes duration were included as covariates.
Significant p-values (<0.05) are highlighted in bold.
[a]The best genetic-effect model for each SNP was estimated based on the smallest Akaike's information criterion (AIC) value. D is dominant model (risk of diabetic complication in heterozygotes plus minor allele homozygotes relative to common allele homozygotes) and R is recessive model (risk of diabetic complication in minor allele homozygotes relative to common allele homozygotes plus heterozygotes).
[*] For these SNPs the dominant model was considered since the rare homozygous genotype was < 3%.
